# The UBC-40 Urothelial Bladder Cancer cell line index: a genomic resource for functional studies

**DOI:** 10.1186/s12864-015-1450-3

**Published:** 2015-05-22

**Authors:** Julie Earl, Daniel Rico, Enrique Carrillo-de-Santa-Pau, Benjamín Rodríguez-Santiago, Marinela Méndez-Pertuz, Herbert Auer, Gonzalo Gómez, Herbert Barton Grossman, David G Pisano, Wolfgang A Schulz, Luis A Pérez-Jurado, Alfredo Carrato, Dan Theodorescu, Stephen Chanock, Alfonso Valencia, Francisco X Real

**Affiliations:** Epithelial Carcinogenesis Group, F BBVA Cancer Cell Biology Programme, CNIO (Spanish National Cancer Research Centre), Madrid, Spain; Servicio de Oncología Médica, Hospital Ramón y Cajal, Madrid, Spain; Structural Computational Biology Group, Structural Biology and Biocomputing Programme, CNIO (Spanish National Cancer Research Centre), Madrid, Spain; Quantitative Genomic Medicine Laboratory, qGenomics, Barcelona, Spain; Departament de Ciències Experimentals i de la Salut, Universitat Pompeu Fabra, Barcelona, Spain; Centro de Investigación Biomédica en Red de Enfermedades Raras (CIBERER), Barcelona, Spain; Institut de Recerca Biomèdica de Barcelona, Parc Científic de Barcelona, Barcelona, Spain; Bioinformatics Unit, Structural Biology and Biocomputing Programme, CNIO (Spanish National Cancer Research Centre), Madrid, Spain; Department of Urology, MD Anderson Cancer Center, Houston, TX USA; Department of Urology, Heinrich-Heine-University, Düsseldorf, Germany; University of Colorado Comprehensive Cancer Center, 80045 Aurora, CO USA; Translational Genomics Laboratory, Division of Cancer Epidemiology and Genetics, National Cancer Institute, Bethesda, USA; Cancer Cell Biology Programme, Centro Nacional de Investigaciones Oncológicas, Melchor Fernández Almagro 3, 28029 Madrid, Spain

**Keywords:** Urothelial bladder cancer, Cell line, Genomics, Mutation, Oncogene, Tumor suppressor

## Abstract

**Background:**

Urothelial bladder cancer is a highly heterogeneous disease. Cancer cell lines are useful tools for its study. This is a comprehensive genomic characterization of 40 urothelial bladder carcinoma (UBC) cell lines including information on origin, mutation status of genes implicated in bladder cancer (*FGFR3, PIK3CA, TP53,* and *RAS*), copy number alterations assessed using high density SNP arrays, uniparental disomy (UPD) events, and gene expression.

**Results:**

Based on gene mutation patterns and genomic changes we identify lines representative of the *FGFR3*-driven tumor pathway and of the *TP53/RB* tumor suppressor-driven pathway. High-density array copy number analysis identified significant focal gains (1q32, 5p13.1-12, 7q11, and 7q33) and losses (i.e. 6p22.1) in regions altered in tumors but not previously described as affected in bladder cell lines. We also identify new evidence for frequent regions of UPD, often coinciding with regions reported to be lost in tumors. Previously undescribed chromosome X losses found in UBC lines also point to potential tumor suppressor genes. Cell lines representative of the *FGFR3*-driven pathway showed a lower number of UPD events.

**Conclusions:**

Overall, there is a predominance of more aggressive tumor subtypes among the cell lines. We provide a cell line classification that establishes their relatedness to the major molecularly-defined bladder tumor subtypes. The compiled information should serve as a useful reference to the bladder cancer research community and should help to select cell lines appropriate for the functional analysis of bladder cancer genes, for example those being identified through massive parallel sequencing.

**Electronic supplementary material:**

The online version of this article (doi:10.1186/s12864-015-1450-3) contains supplementary material, which is available to authorized users.

## Background

Urothelial bladder cancer (UBC) has a high incidence, with 133,696 new cases and 51,056 deaths from UBC in Europe in 2011 [1] and a high prevalence due to the fact that it is commonly an indolent disease. UBC has a higher incidence in males than in females (3:1) and it is the fourth most common cancer in men. Age, smoking, chlorination byproducts, and occupational exposures are the major risk factors [2].

UBC displays a high level of clinical and pathological heterogeneity. Morphologically, tumors can show papillary vs. solid growth patterns. A clinically relevant issue is the level of invasion of the bladder wall: tumors are classified as non-muscle invasive (NMIBC, Ta, carcinoma *in situ*, and T1) or muscle-invasive (MIBC, ≥T2). The majority of patients (ca. 70%-80%) present with papillary NMIBC, most of whom have a good prognosis. Patients with high-grade NMIBC, and those with MIBC, have an aggressive disease that can lead to patient’s death, emphasizing the need to better classify these tumor subgroups.

Approximately 70% of NMIBC harbour activating mutations in *FGFR3*, the main oncogene involved in UBC [3-5]. *PIK3CA* mutations occur in 15% of UBC, often in association with *FGFR3* mutations [6]. An additional 10% of tumors have mutations in *RAS* genes, mutually exclusive with *FGFR3* mutations [7]. MIBC tend to have a low frequency of mutations in *FGFR3* (10%) and develop predominantly through the inactivation of the P53 and RB pathways [4,8,9]. Unlike NMIBC, these tumors are genomically unstable [4,10,11]; several studies have reported the most commonly gained and lost regions [11,12]. *TERT* promoter mutations occur in >70% UBC, regardless of stage/grade [13].

Tumor cell lines are invaluable research tools. They are readily amenable to experimental manipulation, providing opportunities for functional analyses and contributing to improved knowledge [14]. Cell lines have proven useful in preclinical pharmacological studies [15] and will be very important to characterize the function of new cancer genes identified through massive parallel sequencing. However, cell lines often fail to faithfully reflect the genetic and phenotypic diversity of primary tumors and do not fully recapitulate their complexity because the stromal and inflammatory components are not represented *in vitro*. In addition, tumor cells may behave differently in vitro due to the lack of interactions with non-neoplastic cells. Therefore, a thorough knowledge of their genotype and phenotype is essential in order to optimize their use while considering their limitations.

Cell lines from primary UBC are commonly used as disease models. It is crucial to identify those lines best suited to answer specific biological questions and to place the studies in the context of patient’s tumors. The genetic make-up of UBC cell lines has been analyzed using array comparative genomic hybridization [16]. High-resolution gene copy number information for 24 UBC lines is published as part of The Cancer Cell Line Encyclopedia [14] but a detailed comparison of mutations, gene copy number changes, and gene expression is not available. Importantly, the NCI-60 panel does not contain any UBC line [15]. Here, we compile high-resolution genomic information on the largest panel of UBC lines analyzed so far and provide a comprehensive overview of their genetic/genomic architecture. In addition, we use the global transcriptomics data to place the cell lines in the context of the recently reported molecular taxonomy of UBC. This will serve as a reference to the bladder cancer research community and will help to select the most adequate cells to answer specific biological questions.

## Results

We report here a detailed genomic analysis of a large set of UBC cell lines in order to improve their use as models for the study of this tumor type. Web resources used are listed in Additional file 1: Table S1. Mutations were assessed for 49 lines, copy number changes were analyzed in 42 lines, and global expression profiles were gathered for 48 lines. For 40 of them (UBC-40 panel), the complete set of analyses is provided. A summary of the literature search, and our own results, is shown in Table 1 and Additional file 1: Table S2.

**Table 1:** **Genetic characterization and copy number analysis of the major oncogenes and tumor suppressor genes involved in UBC cell lines**

**Name**	**Source**	**Grade**	**Sex**	***FGFR3***	***PIK3CA***	***HRAS***	***KRAS2***	***NRAS***	***TERT***	***INK4A*** **CN status**	***TP53*** **(Mutation/CN)**	**Genome instability group**
**253J**	UCC	G4	F	WT^1,4^	E545G^2,4^	WT^4^	WT^1^	WT^4^	WT^11^	HD ^1,4^	WT/N^3^	Intermediate
**5637**	UCC	G2	M	WT^1,4^	WT^1,4^	WT^1,4^	WT^4^	WT^1^	Mut^11^	WT^1,4^	c.839G > C/N^1,2,3^	Intermediate
**575A**	UCC	G3	M	WT^1,4^	WT^4^					WT	WT/LOH^1^	Intermediate
**639V**	UCC	G3	M	WT^1,4^/R248C^2^	A1066V^1,2,4^	WT^1,4^	WT^1^/G12D^2^	WT^1/^H131R^2^	Mut^11^	LOH^4^	c.743G > A/N^1,2,3^	High
**92-1**	UCC	G3	F	WT^1,4^	WT^4^	WT^4^	WT^4^	WT^4^	Mut^11,12^	WT^6,4^	cd 158, 162, 228, 280 & 294/N^6,8^	Intermediate
**96-1**	UCC	G2/3	M	WT^1,4^	WT^4^	WT^4^	WT^4^	WT^4^	Mut^11,12^	HD^6^	cd 175/N^6,8^	Intermediate
**97-1**	UCC	G1/2	M	WT^1,4^	WT^4^	WT^4^	WT^4^	WT^4^	WT^11^	HD^6^	WT/LOH^6,8^	LOW
**97-18**	UCC	G3	Y	WT^1,4^	WT^4^	WT^4^	WT^4^	WT^4^	Mut^11,12^	LOH^4^	cd 220/LOH^8^	High
**97-24**		G3	Y	WT^1,4^	WT^4^	WT^4^	WT^4^	WT^4^	Mut^11,12^	WT^4^	cd 275/N^8^	High
**97-7**	UCC	G2/3	Y	S249C^1^	WT^4^	WT^4^	WT^4^	WT^4^	Mut^11^	WT4	cd 128/N^8^	High
**BC61**	UCC	G2	Y	G372C^4,10^	WT^4^	WT^4^	WT^4^	WT^4^		WT4	WT/N	Low
**HT1197**	UCC	G4	M	S249C^1,4^	E545K^1,4^	WT^1,4^	WT^1^	WT^1^/Q61R^4^	Mut^11,12^	WT^1^	WT^1^/c.1094A- > G^3^	
**HT1376**	UCC	G3	F	WT^1,4^	WT^1,4^	WT^1,4^	WT^1^	WT^1^	Mut^11^	WT^1,4^	c.749C > T/LOH^1,2,3^	Low
**HU456**		G1	M			WT^4^	G12S^4^	WT^4^	WT^12^	HD^4^	WT/N^7^	Intermediate
**J82**	EC	G3	M	WT^1^/K652E^2,4^	P124L^1,2,4^	WT^1,4^	WT^1^	WT^1^	Mut^11^	WT^1,4^	c.960G- > C&c.820G- > T&c.811G- > A&c.783_919del137/N^1,2,3^	Intermediate
**JON**	UCC			WT^1^/S249C^1^	WT^4^	WT^4^	WT^4^	WT^4^	Mut^11^/WT^12^		Mut^4^	
**KK47**		G1	M			WT^4^	WT^4^	WT^4^	WT^12^	WT^4^	N	High
**LGWO1 G600**				WT^1,4^	WT^4^	WT^4^	G12C^4^	WT^4^	WT^12^	HD^4^	LOH	Low
**MGH-U3**	UCC	G1	M	Y375C^4^/Y373C^1^	WT^4^	WT^4^	WT^4^	WT^4^	Mut^11,12^	HD^4^	WT/N^4^	Low
**MGH-U4**	focal severe urothelial atypia	G1	M	WT^1,4^	H1047R^4^				Mut^12^	HD^4^	WT/N^4^	Low
**PSI**	UCC	G3	M			WT^4^	WT^4^	WT^4^	Mut^12^		WT^7^	
**RT112**	UCC	G2	F	WT^1,2,4^/Amp^4^ */FGFR3-TACC3* fusion ^13^	WT^1,4^	WT^1,4^	WT^1^	WT^1^	Mut^11,12^	HD^1,4^	c.743G > A&c.548C- > G/LOH^1,2,3^	Low
**RT4**	UCC	G1	M	WT^1,4^/Amp^4^/*FGFR3-TACC3* fusion ^13^	WT^1^	WT	WT^1^	WT^1^	Mut^11,12^	HD^1,4^	WT/LOH^1,3^	Low
**SCaBER**	SCC		M	WT^1,2,4^	WT	WT^4^	WT^4^	WT^1^	Mut^11,12^	LOH^4^	c.329G > T/LOH^2,3^	Intermediate
**SW-1710**	UCC		F	WT^1,2,4^	WT^1,4^	WT^1,4^	WT^1^	WT	Mut^11,12^	HD^1^	c.817C > T/LOH^1,2,3^	High
**SW-800**	UCC		M	WT^1,4^	WT^4^	WT^4^	WT^4^	WT^4^	Mut^12^	HD^4^	WT/N^4^	Low
**SW-850**				WT^4^	WT^4^	G12V^4^	WT^4^	WT^4^				
**SW-780**	UCC	G1	F	WT^1,2^/S773F^2^ */FGFR3-BAIAP2L1* fusion ^13^	WT^1,^	WT^1^	WT^1^	WT^1^	Mut^12^	HD^4^	WT/N^1^	Low
**T24**	EC	G3	F	WT^1,4^	WT^1,4^	G12V^1,4^	WT^1^	WT^1^	Mut^11,12^	WT^1^/LOH^4^	c.378C > G/N^1,3^	Low
**TCCSUP**	UCC	G4	F	WT^1,4^	E545K^1^	WT^1,4^	WT^1^	WT^1^	Mut^11,12^	WT^1^	c.1045G > T/LOH^1,3^	Intermediate
**UM-UC-1**	UCC-LN	G2	M	WT^1^	WT^4^	WT^4^	WT^4^	WT^4^		HD^4^	c.454C- > T/LOH^2,3,5^	Intermediate
**UMUC- 2**	UCC	*CIS*	M	WT^1^					Mut^12^		WT^5^	
**UM-UC-3**	UCC		M	WT^1,4^	WT^1,4^	WT^1,4^	G12C^1,2,4^	WT^1^	Mut^11^	HD^1^/WT^4^	c.338 T > G/N^1,3,5,9^	High
**UM-UC-4**	UCC-LC		F	WT^4^	WT^4^					WT^4^	LOH	High
**UM-UC-5**			F	WT^4^	E545K^4^	WT^4^	WT^4^	WT^4^	Mut^12^	HD^4^	LOH	Intermediate
**UM-UC-6**	UCC		M	WT^1^/R248C^4^	E545K^4^	WT^4^				HD^4^	WT/LOH^1,5,9^	Low
**UM-UC-7**			M	WT^4^	WT^4^	WT^4^			Mut^12^	WT^4^	LOH	Intermediate
**UM-UC-9**	UCC			WT^4^	WT^4^	WT^4^			Mut^12^	LOH^4^	Mut/LOH^5,9^	Intermediate
**UM-UC-10**	UCC			WT^4^	WT^4^	WT^4^	WT^4^	WT^4^	Mut^12^		Mut^5^	
**UM-UC-11**	UCC			WT^4^	WT^4^	WT^4^	WT^4^	WT^4^	Mut^12^	HD^4^	WT/N^5^	High
**UM-UC-12**	UCC		Y	WT^4^	WT^4^	WT^4^	WT^4^	WT^4^		WT^4^	N	High
**UM-UC-13**	UCC-LN		Y	WT^4^	WT^4^	WT^4^	WT^4^	WT^4^	Mut^12^	LOH^4^	Mut/N^5^	High
**UM-UC-14**	UCC		Y	S249C^1^	WT^4^				Mut^11,12^	HD^4^	Mut/LOH^5,9^	Low
**UM-UC-15**	UCC			Y375C^4^	E545K^4^	WT^4^	WT^4^	WT^4^	Mut^12^			
**UM-UC-17**				S249C^4^	WT^4^					HD^4^	LOH	Intermediate
**UM-UC-18**				WT^4^	WT^4^	Q61K^4^	WT^4^	WT^4^	Mut^12^	WT^4^	N	High
**VM-CUB-1**	EC	G2	M	WT^1^	WT^1^/E542K + E674Q^2^	WT^1^	WT^1^	WT^1^	Mut^11,12^	c.322G > C^1^/LOH^4^	c.524G > A&c.378C- > G/LOH^1,23^	High
**VM-CUB-2**	EC		M	WT^1,4^	WT^4^	WT^4^	WT^4^	WT^4^	Mut^11^	HD^1,4^	c.473G- > T&c.488A- > G/LOH^3^	High
**VM-CUB-3**	EC	G3	M	WT^1,4^	E545K^4^	WT^4^	WT^4^	WT^4^	Mut^11^	HD^4^	c.833C- > T/N^3^	Low

All TERT mutations were -124 bp(G > A) except the line marked as #-57 bp(T > G).

Amp, amplification; WT, wild type; Mut, mutant; LOH, loss of heterozygosity; HD, homozygous deletion; N, copy number neutral; Y, Y chromosome detected.

^1^COSMIC database; ^2^CCLE database; ^3^IARC database1, COSMIC database; 2, CCLE database; 3, IARC database; ^4^our data; ^5^Specific *TP53* mutation is not specified. [35]; ^6^ *TP53* mutation determined by expression analysis [36]; ^7^[45]; ^8^specific mutation not reported [46], ^9^[47]; ^10^[48]; ^11^[49]. ^12^[13]. ^13^[17].

### The genomic architecture of UBC lines

**Figure 1:**
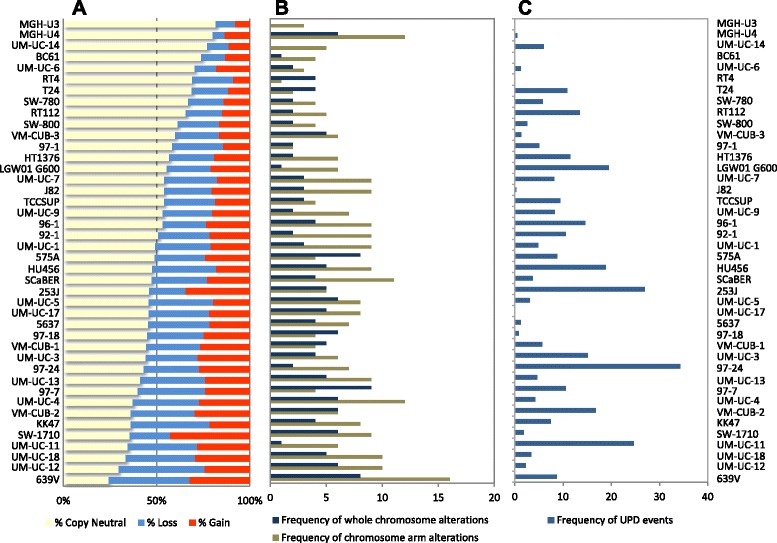
Genomic alterations in cell lines included in the UBC-40 panel. **(A)** Fraction of the genome altered (gains and losses). **(B)** Whole chromosome and whole chromosome arm alterations in the cell lines. **(C)** Fraction of the genome affected by UPDs.

Information on 902,103 autosomal probes covering 2,787 Mb of the genome was analyzed using the waviCGH web server. All of these probes were called as altered (copy number lost or gained) in ≥10 cell lines. An average of 1349 Mb (510-2111 Mb) were altered across the panel: 592 Mb were gained (219-1189 Mb) and 757 Mb were lost (180-1291 Mb). The line showing the lowest fraction of the (autosomal) genome altered was MGH-U3 (510 Mb, 18.3% of the covered genome). 639V cells showed the highest fraction of gains/losses: 2111 Mb (75.8%) (Table 1, Figure 1). The remaining lines showed variable fractions of the genome altered over a continuum; no discrete categories could be identified (Figure 1). Losses were more frequent than gains: an average 28% of covered genome was lost as compared to 21% gained (P = 0.0005). Most cell lines showed loss or gain of multiple whole chromosomes (Figure 1).

### Alterations in oncogenes and tumor suppressors

**Figure 2:**
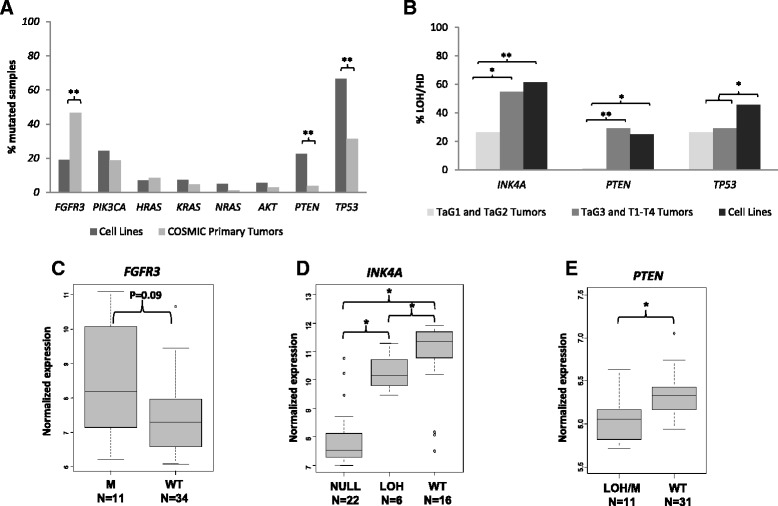
Alterations in the most relevant oncogenes and tumor suppressors involved in UBC. **(A)** Comparison of mutation frequency in UBC lines and tumors. **(B)** Frequency of LOH or homozygous deletion of tumor supressor genes in UBC lines and tumors. **(C)** Association between *FGFR3* mutation status and mRNA expression. **(D)** Association between *INK4A* copy status and mRNA expression. **(E)** Association between *PTEN* copy status and mRNA expression. HD = Homozygous deletion, LOH = Loss of heterozygosity, WT = Wild type.*P < 0.05 **P < 0.01.

Table 1 shows the mutational status of UBC-relevant oncogenes and tumor suppressors. Figure 2A and Additional file 1: Table S3 summarize these results and compare mutation prevalence in cell lines and in primary UBC using information retrieved from the COSMIC database. *FGFR3* mutations were significantly less frequent in cell lines than in tumors (20% vs. 46%, P = 1.9x10^-4^). RT112 and RT4 cells exhibited amplification of a 75 and 79 Mb region, respectively, encompassing *FGFR3* and part of the neighboring *TACC3*. FGFR3 mRNA expression was higher in *FGFR3*-mutant lines (P = 0.09) (Figure 2C). These two lines, and SW-780, have recently been shown to harbour activating translocations involving *FGFR3* [17]. *PIK3CA* mutation frequency was similar in lines and UBC tissues (24% vs. 19%, P = 0.3). Five of 45 lines (11%) harbored a mutation in both *FGFR3* and *PIK3CA*, comparable with the frequency in COSMIC UBC tissues (16%, P = 0.6). Mutations in *HRAS* (7%), *KRAS* (8%), *NRAS* (5%), and *AKT1* (5%) were less frequent (Table 1, Figure 2A, and Additional file 1: Table S3). UM-UC-7 demonstrated amplification of a 7.4 Mb region including *KRAS*. There were no amplifications in *PIK3CA*, *HRAS,* or *NRAS*.

To assess the status of key tumor suppressors (*INK4A*, *PTEN*, and *TP53*) both mutations and genomic losses were considered (Table 1 and Figures 2A and B). VM-CUB-1 was the only line harboring a point mutation in *INK4A;* gene losses were present in 63% of cell lines, including both loss of heterozygosity (LOH) (n = 7) and homozygous deletions (HD) (n = 20). INK4A mRNA expression was significantly lower in lines with LOH (defined as gene copy number loss) or HD than in wild type lines (Figure 2D). As of *PTEN*, 23% and 19% of the lines harboured mutations or LOH. J82 and UM-UC-3 had a *PTEN* mutation and a partial HD. 639V, T24, and UM-UC-9 harboured a missense mutation and retained a wild type allele whereas 5637, RT4, and SW-780 were wild type and showed LOH. Cell lines with LOH or mutant *PTEN* had a significantly lower expression of PTEN mRNA than wild type lines (Figure 2E). *PTEN* mutations were also significantly more frequent in cell lines than in tumor tissues (23% vs. 4%, P = 1.04x10^-4^). Regarding *TP53*, mutations were significantly more frequent in cell lines than in tumors (66% vs. 31%, P = 2.7x10^-6^). LOH was found in 47% of the lines.

Figure 2B compares the frequency of tumor suppressor gene losses in cell lines and tissues analyzed using the same assay platform (n = 49), categorized as non-aggressive vs. aggressive. Gene loss in cell lines and aggressive tumors was comparable (P = 0.64). However, non-aggressive tumors showed a lower frequency of alterations as compared to cell lines (P = 0.02). The frequency of *INK4A* and *PTEN* loss was similar in cell lines and tumors (P = 0.3) but the frequency of *TP53* LOH was higher in cell lines (47% vs. 28%, P = 0.06).

### Original tumor grade, oncogene/tumor suppressor status, and genomic instability

The grade of the original tumor from which 27 lines were isolated was available (Additional file 1: Table S2). Genomic instability, assessed as the size of the genome with copy number alterations, was compared in samples harbouring - or not - mutations in UBC oncogenes and tumor suppressor genes.

In agreement with the genomic analyses of tumors, *FGFR3* mutant lines showed lower genomic instability (genome altered: 1024 ± 461 Mb vs. 1402 ± 349 M, P = 0.06, Wilcoxon). By contrast, *TP53* mutant lines showed higher genomic instability (genome altered: 1381 ± 366 Mb vs. 1023 ± 433 Mb, P = 0.04) (Additional file 2: Figure S1 and Additional file 1: Table S4). Cell lines isolated from low-grade tumors (G1/G2) tended to be more stable than those isolated from high-grade tumors (G3/G4) (Additional file 2: Figure S1). Similar tendencies were observed when using 3 different metrics to assess genomic instability (total size of the genome altered, fraction of probes altered, or number of altered segments identified; see methods section). *FGFR3* mutant lines tended to fall within the genomically stable group whereas *TP53* mutant and high-grade lines tended to fall within the genomically unstable-high group (Additional file 1: Table S5).

### Copy number changes involving whole chromosomes/whole chromosome arms

Because distinct mechanisms lead to alterations in whole chromosomes or chromosome arms and to interstitial changes, these were assessed separately. Most cell lines showed losses and gains of multiple whole chromosomes/whole chromosome arms (Figure 1, Table 2, and Additional file 1: Table S6). Chromosomes most frequently gained were chr.20 (41%), chr.7 (23%), chr.21 (20%), and chr.5 (11%). The chromosome arms most frequently gained included 5p (45%), 8q (39%), 3q (34%), 7p (18%), 9q (18%), 1q (18%), 20q (16%), 20p (14%), and 9p (11%). Chromosomes most frequently lost were chr.4 (34%), chr.1 (27%), chr.21 (25%), chr.15 (20%), chr.22 (20%), chr.13 (16%), and chr.16 (16%). The most common arm losses included 8p (52%), 18q (25%), 3p (25%), 9p (23%), 17p (20%), and 2q (18%).

**Table 2:** **Frequency of whole chromosome or chromosome arm alterations in UBC lines (n = 42)**

	**Chromosome**	**1**	**2**	**3**	**4**	**5**	**6**	**7**	**8**	**9**	**10**	**11**	**12**	**13**	**14**	**15**	**16**	**17**	**18**	**19**	**20**	**21**	**22**
**Losses**	**Whole chromosome**	5	9	2	34	2	2	0	0	7	11	0	7	16	7	20	16	2	27	9	2	25	20
	**p-arm**	9	5	25	9	2	5	5	52	23	9	14	5	7	0	2	2	20	5	7	2	0	0
	**q-arm**	2	18	0	11	9	5	0	0	5	9	0	2	2	0	2	0	0	25	7	0	0	0
**Gains**	**Whole chromosome**	0	0	0	0	11	2	23	2	7	0	0	2	7	7	2	0	7	0	2	41	20	7
	**p-arm**	2	9	0	2	45	9	18	0	11	7	7	9	0	0	0	0	5	5	2	14	2	0
	**q-arm**	18	2	34	0	2	9	7	39	18	2	7	5	7	2	0	7	5	0	5	16	0	0

### Recurrent focal copy number alterations across cell lines

**Figure 3:**
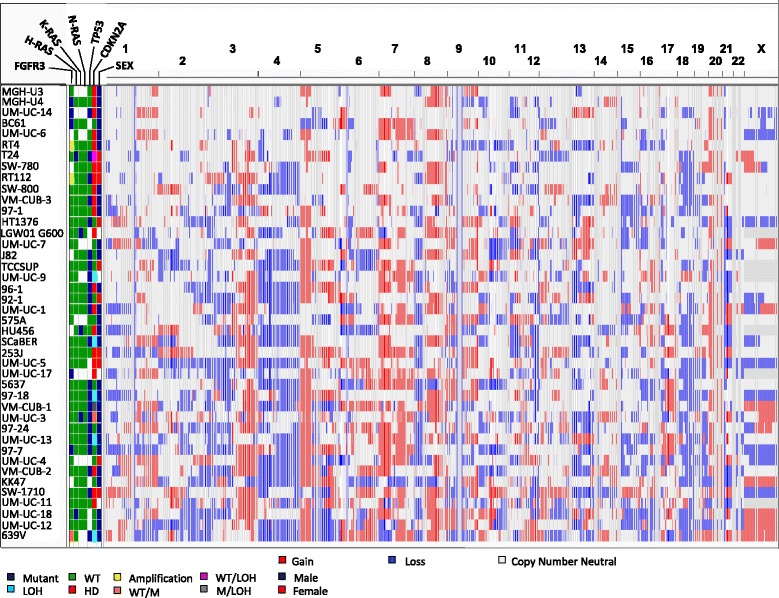
Genome wide copy number calls across the genome in UBC lines. Gender and oncogene/tumor suppressor status are also annotated.

Figure 3 shows copy number calls of individual probes for each line; Table 3 shows statistically significant minimal common regions (MCRs) identified using waviCGH, a permutation-based method [18]. Altogether, 21 statistically significant (FDR <0.05) MCRs were identified (11 gained and 10 lost), ranging from 1.6 kb-156 Mb in size (gains: 211 Kb-56 Mb; losses: 1.6 Kb-156 Mb). Six MCRs almost entirely covering chromosome 4 were identified; some MCRs overlapped with whole chromosome or chromosome arm changes, such as gains in 1q, 3q, 5p, 7, and 21 and losses in 3p, 4, and 15. Other MCRs included gains at 11p15 and losses at 6p22.1-6p22.2, 10q23.33, and 13q33.3 (Tables 2 and 3, Figure 3, and Additional file 1: Table S6). All recurrent focal losses were hemizygous.

**Table 3:** **Focal gene copy number alterations and minimal common regions (MCR) identified using waviCGH in UBC lines**

**Variation type**	**Chr**	**Length (KB)**	**Number of probes**	**Adjusted p value**	**Frequency (% Cell Lines)**	**CytoBand (Probe boundaries)**	**Number of genes**	**Previously described**
**GAIN**	1	15995	4751	0.05	35	1q25.3-1q31.3 (rs502870- rs590258)	164	Tumors: 1q gain, 1q24.2 and 1q23 amplification SETDB1 (1q21)
	1	1517	695	0.05	38	1q32.2 (rs1126573- rs2494606)	0	Novel
	3	4084	1623	0.02	35	3q21.3-3q22.1 (rs34267791-rs6439205)	110	3q21.3 tumors
	5	49651	16712	0.05	35	5p13.1-5p12 (rs28538767- rs36047540)	609	Novel
	7	55653	22123	0.01	42	7p15.2-7p11.2 (rs4096522- rs10280445)	848	7p11.2 amplification high grade tumors
	7	840	347	0.05	38	7q11.23 (rs11544049- rs2074666)	22	Novel
	7	21068	7166	0.05	38	7q21.3-7q31.2 (rs10953601- rs10246291)	847	7q22.1 and 7q32 amplification in tumors
	7	3252	1290					
	7	24003	7258					
	7	211	96	0.05	38	7q33 (rs10260266- rs3807337)	5	Novel
	11	3044	1510	0.05	35	11p15.5-11p15.4 (rs4029252- rs7103275)	152	Tumors: 11p loss
**LOSS**	3	22040	8521	0.04	46	3p21.1-3p14.2 (rs4927997- rs13075591)	240	Tumors/cell lines
	4	310	78	0.01	44	4p16.3 (rs10446889- rs13137548)	15	Tumors/cell lines
	4	665	272	0.05	40	4p16.3-4p15.1 (rs1728273- rs11930062)	379	Tumors/cell lines
	4	4561	2058					
	4	22491	7467					
	4	1839	399	0.05	40	4p13-4q35 (rs7665332- rs13124496)	2003	Tumors/cell lines
	4	155726	47697	0.01	44			
	6	4227	3338	0.01	42	6p22.1 (rs498548- rs9468692)	267	MHC region
	10	640	424	0.03	40	10q23.33 (rs17110194- rs11188277)	17	Tumors/cell lines
	13	1.60	5	0.03	38	13q33.3 (rs3093749- rs1805385)	1	Tumors/cell lines

Eight regions were amplified in ≥3 cell lines (81 Kb-73 Mb) (Additional file 2: Figure S2 and Table 4), mostly in chromosomes or chromosome arms lacking high frequency alterations. Six of them have previously been described as gained/amplified in UBC tissues but not in cell lines; another region at 12p11.22-12q13.13 is novel to both tumors and cell lines.

**Table 4:** Regions of genomic amplification in UBC lines*

**Chr**	**Length (KB)**	**Number of probes**	**Frequency (% Cell Lines)**	**Cytoband**	**Number of genes**	**Previously described**
1	81	42	7-11	1p36.22	6	Tumors: gain
3	73857	848	7	3p25.2-3p12.1	1052	Cell lines: 3p loss
6	3837	1516	7-14	6p22.3	42	Tumors E2F3
11	20016	1462	7-18	11p11.12-11q13.4	782	Tumors: 11p loss
11	29380	1165	7-11	11q22.1-11q24.2	616	Tumors: 11q loss
12	21857	87	7-9	12p11.22-12q13.13	452	Novel
14	3856	1157	7	14q21.2	26	Tumors: 14q loss
17	55108	1298	16-18	17p11.2-17q25.1	1448	Tumors: 17p loss

*Statistically significant MCR.

### X chromosome analysis

Data regarding the X chromosome could be evaluated in 37 lines (9 female and 28 male). Large structural alterations were rare: 6 lines showed complete loss of Xp whereas 3 lines showed almost complete gain of Xq. No significant MCRs were identified although peak gains were seen at Xp22.2, Xp11.4, Xp11.23, Xq11.2, Xq12, and Xq25 and peak losses at Xq21.31 and Xp21.3-21.1 (Additional file 2: Figure S3).

### Uniparental disomies (UPD)

**Figure 4:**
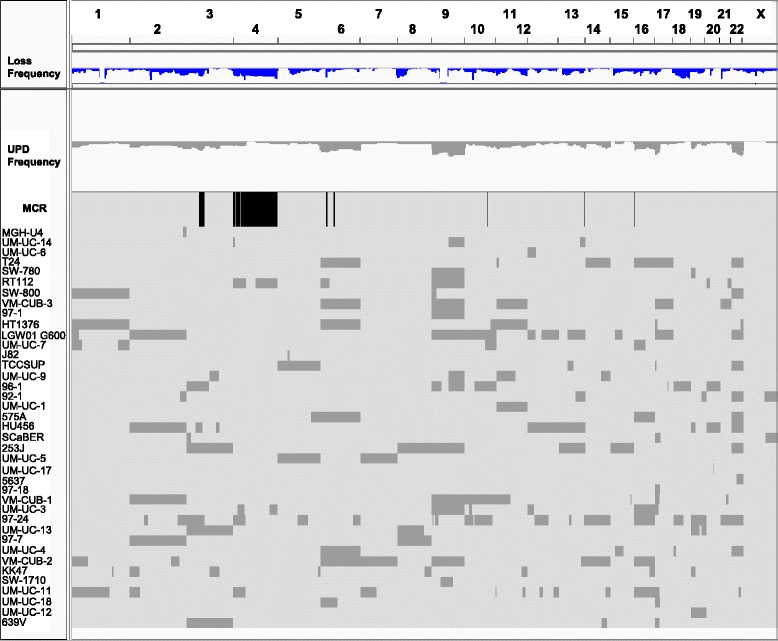
Genome wide assessment of regions showing UPD identified in UBC lines.

Autosomes were analyzed for the presence of UPD (Figure 4 and Additional file 1: Table S7), defined as copy number neutral or amplified regions showing LOH. Examples of different categories of UPD events are shown in Additional file 2: Figure S4A-F. Overall, 195 UPD events were identified in 40 lines: UPDs were absent from BC61, RT4, and MGH-U3. Interestingly, these lines are among those showing lower fraction of the genome altered (Figure 1). All autosomes displayed ≥1 UPD event in ≥2 lines. The median number of UPD events per line was 4; cell line 97-24 showed 22 UPD events. Focal UPDs were the most common event (n = 91), ranging in size from 2-129 Mb. There were 51 UPDs involving whole chromosomes and 39 UPDs of a whole chromosome arm. UPDs involving whole chromosomes were most common in chr. 9, 17, and 22.

Many UPD events occurred in regions that are lost in other lines, supporting the occurrence of tumor suppressors therein. For example, region 3p21.1-3p14.2, lost in 46% of lines, coincides with a recurrent UPD. In addition, 4 lines show UPD of chr.3 (639V, 253J, and UM-UC-13) or 3p arm (96-1). The number of UPD and their total genome size correlated with the genome size affected by copy number alterations (p = 0.08 and p = 0.09) (Additional file 2: Figure S5). *FGFR3* mutant cell lines had significantly fewer UPD events and overall size of the genome affected by UPD than wild type lines, supporting that UPD associates with aggressiveness (Additional file 2: Figure S6).

### Gene-level analysis of copy number alterations

**Figure 5:**
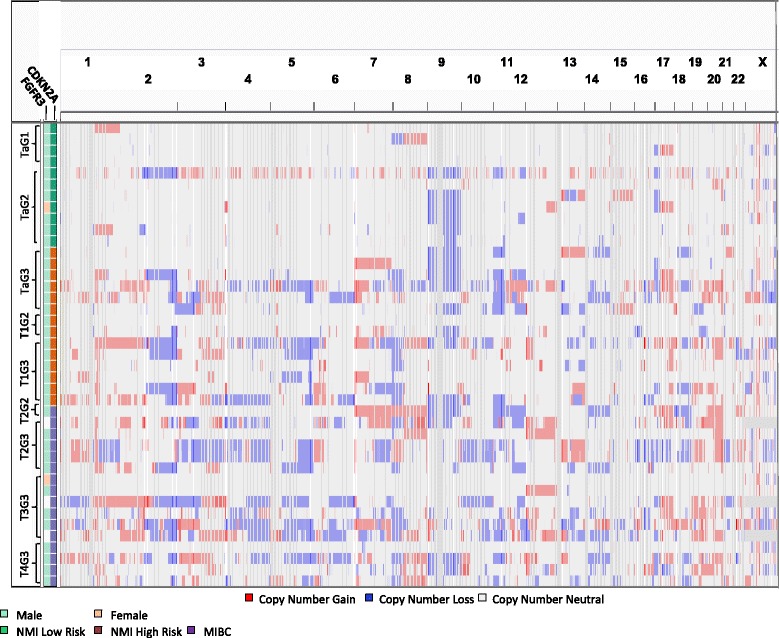
Genome wide copy number calls in primary bladder tumors (n = 49) with T and G annotation.

Gene copy number data was newly generated from 19 low-risk and 30 high-risk primary UBC. Some gains (5p, 8q, 17q, and whole chr.20) and losses (5q, 8p, 17p) occurred with similar frequency in lines and tumors (Figures 1B, and 5, Table 2, and Additional file 1: Table S6).

Tumors typically showed whole chr.9 loss, likely targeting multiple tumor suppressors (i.e. *INK4A*, *PTCH1*, and *TSC1*) whereas the cell lines had a high frequency of both gains and losses of chromosome 9, often in association with UPD affecting either the whole chromosome or its q-arm (Figure 4). Partial chr. 9 UPDs were found in several cell lines although there was no overlap among the regions affected. Chr.19 and chr.22 were more frequently lost in lines whereas they were more often gained in tumors (Figure 5).

### Comparison of gene copy number alterations and expression

The complete expression dataset is provided in Additional file 1: Table S8. The 8 regions amplified in ≥3 cell lines (Additional file 1: Table S9) include 825 protein-coding genes with microarray expression information; 396 of them had a higher average expression in lines with gains/amplifications vs. those without them. This difference was statistically significant for 51 genes (Additional file 1: Table S9). Among them are *CDKAL1* (CDK5 regulatory subunit associated protein like 1, 6p22.3; 4-fold differential expression, p < 0.05), *ASRGL1* (11q13.4), *ATP2B4* (1p12), *ITGA3*, *PRPSAP2*, and *C17orf39* (17p11.2), all with a fold-change difference between amplified and non-amplified tumors of 1.5-1.9 (p < 0.05).

Gene expression information for 334 genes in the 10 lost regions was available; 225 had a lower average expression in cell lines with loss vs. those without loss. Of them, 28 showed statistically significant differential expression, including *ANXA10* (4q32.3; 4.8-fold), *ARAP2* (4p14; 2-fold), *CDS1* (4q21.23; 2.2-fold), and *PTPRG* (3p14.2; 1.6-fold) (Additional file 1: Table S9).

### Genomic analyses of new genes involved in UBC identified through exome sequencing

We analyzed copy number, UPD status, and expression of new driver UBC genes (Additional file 1: Tables S10 and S11) identified through exome sequencing [19-24]. Several of them are in genomic regions with either whole chromosome/chromosome arm gain/loss. LOH, and gains were more common than UPD (average 10 vs. 4 events per gene). *PDZD2* and *CSMD3* were often gained (41%, 64% and 52% respectively) whereas *ANK2*, *FAT4* and *MLL* were often lost (55%, 55% and 50% respectively); *MLL* is on chromosome 11 - which is not frequently altered in UBC - and is significantly under-expressed in cell lines with LOH (Additional file 1: Table S11). *TSC1* showed both gains (52%) and UPD (29%); a similar pattern was observed for other tumor suppressor genes. *TP53* and *EP300* were affected by both LOH and UPD.

### UBC cell lines represent molecularly defined bladder cancer subtypes

**Figure 6:**
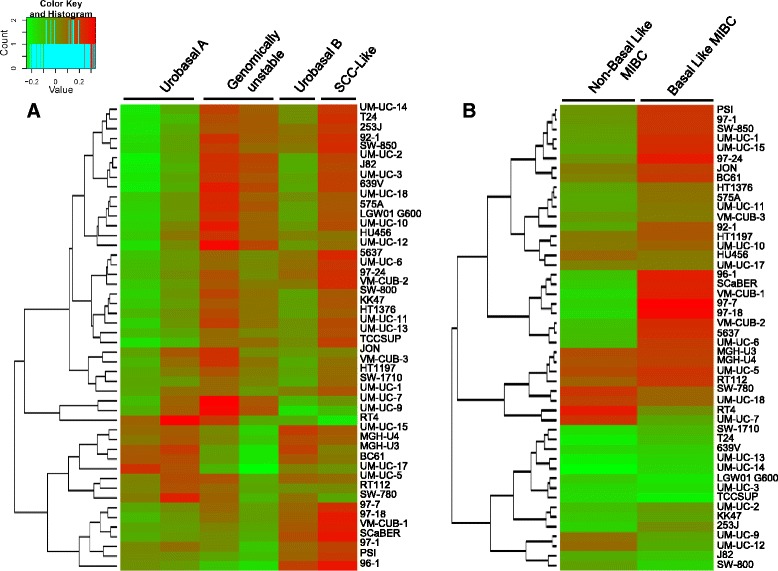
Clustering of UBC lines according to the expression of gene signatures used to molecularly classify primary tumors. **(A)** Cell lines displaying expression patterns of the “Urobasal A”, “Urobasal B”, and “SCC-like” by Sjodahl et al [12]. The “Genomically Unstable” category is poorly defined. **(B)** Cell lines displaying expression signatures of “Basal-like” or “Non Basal-like” tumors according to the classification of Rebouissou et al. [24].

We applied the UBC molecular classifier based on gene expression defined by Sjodahl et al [12] to identify lines most representative of the taxonomical groups proposed. Figure 6A shows that cell lines could be adscribed to the “Urobasal A”, “Urobasal B”, and “SCC-like” classifiers (Additional file 1: Table S12). The “Genomically Unstable” group was most commonly represented among the lines. Rebouissou et al. have recently reported on a 40-gene basal-like signature [25]. We have applied their 40-gene classifier to the cell line dataset and identify 4 major groups: lines with a predominant enrichment in the “Basal-like” signature; lines with a predominant enrichment in the “Non basal-like” signature; lines with enrichment of both signatures; and lines in which none of the signatures is enriched (Figure 6B and Additional file 1: Table S13). In agreement with the gene mutation/copy number change data indicating that cell lines are biased towards a more aggressive type, the “Non Basal/Luminal” phenotype is less represented among the available established cell lines.

## Discussion

This is the most comprehensive analysis of the genomic landscape of UBC lines reported to date, including mutation, copy number, and expression data for a panel of UBC lines. As we have complete copy number and gene expression data for 40 of them, we have named this dataset UBC-40.

We provide detailed information on the source of the cell lines used in order to avoid “mistaken identity”. When surveying the literature, there are conflicting reports regarding the mutation status of some of the genes studied possibly due to cell contamination or mislabeling; similar problems have been reported with lines from the NCI-60 panel [26]. For example, MGH-U1/EJ and some subcultures of J82 are derived from T24 [27] and KU7 was cross-contaminated with HeLa [28]. Cell lines extensively cultured in different laboratories may have evolved independently and ultimately acquired mutations or genomic changes absent from the original line [29]. Nevertheless, fundamental features of these cell lines show stability and have allowed their extensive use as models of UBC in a wide variety of studies; this is the case of FGFR3-dependent RT112 cells [30]. The clustering shown in Figure 6 allows to propose, using two independent classifiers, prototype cell lines of papillary/luminal tumor phenotypes (i.e. RT4, UM-UC-7), and Basal-like tumors (well represented by several cell lines). Interestingly, a few cell lines display enrichment in both Basal and Non-Basal gene signatures (i.e. RT112 and MGH-U3, both of which are known to be FGFR3-driven). This information will be useful for selecting the best models to address specific functional studies.

Most of the lines analyzed here were generated many years ago, have been extensively passaged, and do not have matched normal tissue - or lymphoblastoid B cells - from the same patient available. Therefore, it is not possible to define the somatic variants they carry, this being the reason why we have not conducted exome sequencing. Consequently, and because the lines available only represent incompletely the spectrum of UBC as it relates to low-grade tumors, renewed efforts should be placed in the establishment of new cell lines and xenografts to facilitate preclinical studies. Improved sample processing, matrigel embedding, and orthotopic implantation, as well as more reliable systems for primary culture and passage [31,32], should contribute to improve efficiency. Furthermore, efforts should be made to biobank non-neoplastic material from the same patients.

The summary data reported here provides an overview and the detailed datasets should serve as a resource to the research community in order to identify which - among these lines - serve best as disease models for specific tumor subtypes. The recent discovery of new driver genes involved in UBC through massive parallel sequencing [19-24] and the information provided here should be useful to select lines appropriate for their functional analysis and for preclinical studies.

## Conclusions

- *TP53-*mutant lines show high genomic instability whereas *FGFR3-* or *PIK3CA-*mutant lines are more genomically stable.

- We have identified for the first time UPD events in UBC lines, pointing to new regions containing putative tumor suppressors.

- We provide novel information on chr. X losses, where new important tumor suppressor genes have been identified (i.e. *KDM6A* and *STAG2*).

- We identify novel regions deserving research as they are frequently altered in UCB lines as well as in primary tumors.

- Some cell lines are more representative of the *FGFR3*-driven tumor pathway (RT112, MGH-U3, 97-7, BC61, RT4, SW-780, and UM-UC-6) whereas others are more representative of the tumor suppressor-driven pathways (5637, 92-1, 96-1, 97-18, 97-24, HT1376, SW-1710, UM-UC-1, UM-UC-13, and VM-CUB-2). We propose that - as has already been done, in part - these cells be used as models of non-aggressive and aggressive UBC, respectively.

- The UBC lines available cover a wide range of tumor genotypes and phenotypes. While they do not fully represent the spectrum of tumors found in patients and are enriched towards a more aggressive genetic architecture, the main genetic pathways involved in UBC are represented in this panel.

- Future efforts should be placed to establish new UBC lines, mainly focusing on less aggressive tumors, as well as collections of patient-derived xenografts.

## Methods

### Literature and web-based search

A literature search was performed to retrieve information regarding sex, histology, and stage/grade of the original tumor from which the cell line was established, as well as the original reference. The mutational status of the main genes involved in UBC (*TERT*, *FGFR3*, *PIK3CA*, *KRAS*, *HRAS*, *NRAS*, *p16/INK4A*, *PTEN*, and *TP53*) was analyzed and complemented/supported through data obtained from COSMIC, CCLE, and IARC public databases [14,33,34]. Information on mutations in *FGFR3*, *PIK3CA*, *KRAS*, *HRAS*, *NRAS*, *PTEN*, and *TP53* in primary UBC tumors with either “papillary” or “carcinoma” histology was retrieved from the COSMIC database. All web resources used in the analysis are listed in Additional file 1: Table S1.

### UBC cell lines

JON, MGH-U4, RT4, SCaBER, SW-800, SW-850, SW-1710, T24, VM-CUB-2, 253J, 639V, 5637, and 575A were purchased from the American Type Culture Collection (Rockville, MD, US); J82, MGH-U3, and RT112 cells were kindly provided by F. Radvanyi (Institut Curie, Paris, France); UM-UC-1, UM-UC-3, UM-UC-4, UM-UC-5, UM-UC-6, UM-UC-7, UM-UC-9, UM-UC-10, UM-UC-11, UM-UC-12, UM-UC-13, UM-UC-14, UM-UC-15, UM-UC-17, and UM-UC-18 were provided by H. B. Grossman (MD Anderson Cancer Center, Houston, TX, US) [35]; HT1197, HT1376, HU456, KK47, PSI, SW-780, UM-UC-2, and VM-CUB-1 were provided by D. Theodorescu (University of Colorado, Aurora, CO); 92-1, 96-1, 97-1, 97-7, 97-18, and 97-24 were generated by C. Reznikoff [36] and provided by M. Knowles (University of Leeds, Leeds, UK); TCCSUP was provided by M. Sánchez-Carbayo (CNIO, Madrid, Spain); BC61 was provided by W. Schulz [37]; and LGWO1 G600 was provided by J. Reeder (U. Rochester, NY). Only Mycoplasma-free cultures were used.

### DNA and RNA isolation from cell lines and tumors

Cells were cultured in RPMI supplemented with 10-20% FBS and were harvested at 70-90% confluence. DNA from cell lines HT1197, HT1376, HU456, KK47, PSI, UM-UC-2, and VM-CUB-1 was isolated in the laboratory of D. Theodorescu; the remaining cell lines were cultured at CNIO. DNA was isolated using the DNAeasy blood and tissue kit (Qiagen) according to manufacturer’s instructions.

Tumor samples (n = 49) came from UBC cases diagnosed with UBC recruited to the Spanish Bladder Cancer/EPICURO study. Informed consent was obtained from study participants in accordance with the Institutional Review Board of the Ethics Committees of participating hospitals that approved the study (IRB Hospital del Mar, ref. 2008/3296/1). The T/G distribution was as follows: Ta (n = 26), T1 (n = 8), T2 (n = 5), T3 (n = 6), and T4 (n = 4); cases were grouped in two categories, non-aggressive (TaG1 and TaG2) (n = 19) and aggressive (TaG3 and T1-T4) (n = 30). Only samples containing >60% tumor cells were used. DNA was isolated using the Puregene kit A (Qiagen) according to the manufacturer’s instructions.

### Mutational analyses

*FGFR3*, *PIK3CA*, *HRAS*, *KRAS*, and *NRAS* hotspot mutational analysis was performed using ABI PRISM® SNaPshot® (ABI) as previously described [38].

### Analysis of gene copy number alterations using the Illumina 1 M Duo array

DNA (1.5 μg) was quantified using picogreen and used for array hybridization. The Illumina 1 M Duo array includes 902,103 autosomal probes and 39,779 probes from sex chromosomes. A total of 43 different cell lines were hybridized to the arrays (GSE64572) Genotypes and R values were extracted using the beadstudio software (version 3.1.3.0) and R values were normalized using the method described by Pounds and co-workers [39]. Log R ratios were calculated using as reference the average R value from 200 blood leukocyte samples from control subjects included in the EPICURO study [40] with the R program version 2.8 [41]. Copy number calls were obtained using the waviCGH software [18]; segmentation and calling were performed using DNAcopy [42] and the probability-based method (CGHcall) [43], respectively. Gene copy number changes were called as follows: -1 = loss (hemi or homozygous), 0 = copy number neutral, +1 = gain and +2 = amplification (defined as ≥5 copies). Minimal common regions (MCRs) were identified using the permutations method in waviCGH which computes a *P*‐value based on a permutation test assuming that the alterations found are randomly located in the genome. Consecutive probes with *P*‐values <0.05 were joined in a common region*.* Focal copy number alterations are those not involving whole chromosomes or whole chromosome arms.

### Gene copy number reproducibility analysis

The experimental reproducibility of the gene copy number analysis was assessed using data from DNA isolated in 2 different laboratories (n = 3) or DNA isolated from different cultures in the same laboratory (n = 5). The absolute call concordance rate was between 79.3 and 96.7%. “Gain/loss” type discordances were very uncommon (0.015-0.03%); most discordances were “gain/no-change” (1.4-7.5%) or “loss/no-change” (1.9-15.8%). The replicate with the highest signal to noise ratio was considered the most accurate and selected for subsequent analysis. A summary of the call concordance rate in replicates is provided in Additional file 1: Table S14.

### Copy number analysis of *FGFR3*, *PIK3CA*, *KRAS*, *HRAS*, *NRAS*, *INK4A*, *PTEN*, and *TP53*

Gene amplification was determined from the copy call results from CGHcall. LOH and HD were determined by combining copy call, B allele frequency (BAF), and genotyping data. Probes with homozygous calls and BAF of either 0 or 1 and a decline in the logR ratio of were classified as LOH and non-called (NC) probes with an abnormal BAF and a decline in LogR ratio were classified as HD. The number of probes representing each gene was: *FGFR3* (n = 22), *PIK3CA* (n = 42), *KRAS* (n = 22), *HRAS* (n = 2), *NRAS* (n = 6), *INK4A* (n = 17), *PTEN* (n = 49), and *TP53* (n = 22).

### Copy number analysis of genes in X chromosome

The gender of the patient from whom the cell lines were derived was not always available. The presence of a Y chromosome was considered as a reliable indication that it was of male origin. This, combined with the data from the literature, was used to select cell lines for which we could perform X chromosome gene copy number analysis. The LogR ratio was calculated with the average R value normalized to a pool of control male or female blood leukocytes from the EPICURO Study. For probes representing the X chromosome (38,016 probes), or those corresponding to sequences present on both the X and Y chromosomes (395 probes), copy number calls were obtained using the waviCGH software as described for autosomes.

### Assessment of genomic instability

Genomic instability was assessed as the fraction of the genome altered (either lost or gained), calculated from call data generated by CGHcall using autosome probes and measured in 3 different ways:

(1) total size of the genome (covered by the Illumina array) altered. The size in base pairs (bp) of the segmented regions altered (lost or gained) was calculated from the start and end position of the segments; (2) fraction of probes altered - proportion of probes showing loss, gain, and amplification; (3) number of altered segments identified - the total number of individual altered segments, lost or gained, was determined from the waviCGH segmented call data.

Cell lines were classified in 3 categories according to fraction of the genome altered (upper, middle, and lower tertile) calculated by the 3 different methods; low/medium/high genomic instability groups were thus identified. We compared the relationship between mutational status in *FGFR3*, *PIK3CA*, *KRAS*, *HRAS*, *NRAS*, *p16/INK4A*, *PTEN*, and *TP53* and the original tumor grade with the fraction of the genome altered, calculated by these approaches. The chi-square test was used to assess the difference between the frequency of mutant vs. wild type genotypes and low vs. high grade cell lines in each of the genome instability groups. The Wilcoxon test was used to assess the difference in the mean of the genome instability variable between mutant/wild type and low/high grade cell lines.

### Uniparental disomy (UPD) detection

Log R ratios from hybridization data were analyzed using the zoo package of the R statistical program in order to identify UPDs [40]. Chromosomal regions with LOH, as determined from the BAF, and an average LogR ratio value around 0 indicate a probable segmental UPD. UPD events were classified into 6 different categories: (1) involving the whole chromosome, (2) involving a whole chromosome arm, (3) focal UPD, (4) focal UPD and segmental duplication, (5) UPD and segmental amplification, and (6) UPD involving almost the entire chromosome with a combined focal deletion.

### RNA expression analyses

We have used previously reported data corresponding to 28 cell lines (GEO: GSE5845) [25] and have generated expression data for 20 additional cell lines (GSE64279). RNA was isolated with Trizol in both experimental batches. For the new cell lines, RNA (500 ng) was amplified, labeled, and used for array hybridization. The Affymetrix U133A array was used in all experiments. Raw expression data from all experiments were normalized using the R library Frozen Robust Multiarray Analysis (fRMA) method [44]. We applied this method as described by the authors for multiple arrays. We read the raw data (CEL files) and used the Random effect model for preprocessing. This model allows us combining data from different batches using the same microarray platform for analysis. Further, to obtain a matrix of gene-level expression values we used the *exprs* function with parameters by default.

### Comparison of gene copy number and expression data

Gene lists were generated for regions identified as significant MCRs in copy number lost, gained or amplified regions. To determine the relationship between gene copy number alteration and expression, correlation analysis was performed for amplified genes by comparing copy number amplified/gained cases vs. copy number neutral/lost cases. For copy number lost genes, a comparison of copy number lost vs. no copy number loss was performed; the difference in expression between the 2 groups was assessed using the Wilcoxon test. The chi-square test was used to compare the distribution of mutation frequencies in cell lines and tumors.

### Gene copy number, UPD analysis, and comparison with expression data of 44 new genes implicated in UBC

Copy number status and UPD of 44 genes recently shown to be involved in UBC were assessed [20,21,23,24]. To determine the relationship between gene copy number alteration and expression, correlation analysis was performed for gained genes by comparing copy number amplified/gained cases vs. copy number neutral/lost cases. For copy number lost genes, a comparison of copy number lost vs. no copy number loss was performed; the difference in expression between the groups was assessed using the Wilcoxon test.

### Molecular classification of cell lines according to expression signatures of primary tumors

A molecular classifier consisting of 1038 genes [12] was used for hierarchical clustering; M. Lauss kindly provided the gene expression values for each signature (Additional file 3) and the classification method (Additional file 4). We processed the data to obtain one expression value for each gene present in the Affymetrix U133A array, as different probes for same gene are present in the platform. For this purpose we used the CollapseDataset tool available in the GenePattern webserver with collapse mode set at maximum. Then, we extracted the common genes present in the centroids and our expression file to calculate the pearson correlation for each centroid to each sample (classification script is provided as Additional file 4) using the R software. Furthermore, hierarchical clustering and heatmap plots were generated with the heatmap.2 function included in the gplots library in R software using Pearson correlation values calculated previously for each centroid to each sample (Additional file 1: Table S12). Expression signatures were re-named as “Urobasal A” (MS1a-b), “Genomically unstable” (MS2a1-2),“Urobasal B” (MS2b2.1) and “SCC-like” (MS2b2.2). The “Infiltrated” phenotype was not considered as it is mainly based on a stromal signature.

The same approach was applied using the centroids provided by Rebouissou et al. [25] to classify cell lines as “basal” or “non-basal” with the respective confidence prediction (Additional file 1: Table S13).

### Data availability

All genomic data reported in this manuscript have been deposited in GEO. Expression data accession number: GSE64279. SNP array data accession number: GSE64572. Any other information of interest to readers can be requested to the authors.

## Additional files

Additional file 1: Table S1. List of web resources used. **Table S2.** Characteristics and frequency of mutations in key oncogenes and tumor suppressors in UBC lines and in urinary bladder tumors. **Table S3.** Frequency of mutations in key oncogenes and tumor suppressors in UBC lines and in urinary bladder tumors. **Table S4.** Relationship between genomic instability, *FGFR3,* and *TP53* mutation status and grade of original tumor. **Table S5.** Chi square analysis of genomic instability of UBC lines, original tumor grade, and *FGFR3* and *TP53* mutation status. **Table S6.** Summary of large gene copy number alterations involving whole chromosomes or whole chromosome arms. **Table S7.** UPD analysis of UBC cell lines. **Table S8.** Affymetrix expression array data of UBC lines included in the study. **Table S9.** Genes in lost and amplified regions and comparison of gene copy number and gene expression data. **Table S10.** Gene copy number changes and UPD events in new driver genes identified recently through exome/whole genome sequencing analysis of UBC tissues. **Table S11.** Comparison of copy number data and expression of 44 new genes implicated in UBC. **Table S12.** Pearson’s correlations according to the gene signature from Sjodahl et al [12]. **Table S13.** Pearson’s correlations according to the 40-gene signature from Rebouissou et al [25]. **Table S14.** WaviCGH call all concordance rate in replicate cell lines. Additional file 2: Figure S1. Association between genomic instability (measured as fraction of the genome altered) (bp)and*FGFR3* mutation status, *TP53* mutation status, and Grade of original tumor from which cell lines were established. **Figure S2.** Genome wide assessment of gene amplifications in UBC lines. **Figure S3.** Log R ratios of X chromosome probe signal in UBC lines and gender origin. **Figure S4.** UPD analysis in UBC lines. **Figure S5.** Relationship between UPD events and copy number alterations. **Figure S6.** Uniparentaldisomies (UPD) and *FGFR3* mutation status. **Figure S7.** Segmented mean Log ratios of probe signals for UBC lines in the Cancer Cell Line Encyclopedia (http://www.broadinstitute.org/ccle/home). Additional file 3:
**Classification script for molecular subtyping of bladder tumors kindly provided by Lauss M with modifications by Carrillo-de Santa Pau E.**
Additional file 4:
**Centroids file kindly provided by Sjödahl G, Lauss M and Höglund M generated in Clin Cancer Res. 2012 Jun 15;18(12):3377-86.**

## Abbreviations

HD: Homozygous deletions
LOH: Loss of heterozygosity
MCR: Minimal common regions
MIBC: Muscle-invasive bladder cancer
NMIBC: Non-muscle invasive bladder cancer
UBC: Urothelial bladder cancer
UPD: Uniparental disomy
