# Erratum to: The UBC-40 Urothelial Bladder Cancer cell line index: a genomic resource for functional studies

**DOI:** 10.1186/s12864-016-3179-z

**Published:** 2016-10-25

**Authors:** Julie Earl, Daniel Rico, Enrique Carrillo-de-Santa-Pau, Benjamín Rodríguez-Santiago, Marinela Méndez-Pertuz, Herbert Auer, Gonzalo Gómez, H. Barton Grossman, David G. Pisano, Wolfgang A. Schulz, Luis A. Pérez-Jurado, Alfredo Carrato, Dan Theodorescu, Stephen Chanock, Alfonso Valencia, Francisco X. Real

**Affiliations:** 1 Epithelial Carcinogenesis Group, F BBVA Cancer Cell Biology Programme, CNIO (Spanish National Cancer Research Centre), Madrid, Spain; 2 Servicio de Oncología Médica, Hospital Ramón y Cajal, Madrid, Spain; 3 Structural Computational Biology Group, Structural Biology and Biocomputing Programme, CNIO (Spanish National Cancer Research Centre), Madrid, Spain; 4 Quantitative Genomic Medicine Laboratory, qGenomics, Barcelona, Spain; 5 Departament de Ciències Experimentals i de la Salut, Universitat Pompeu Fabra, Barcelona, Spain; 6 Centro de Investigación Biomédica en Red de Enfermedades Raras (CIBERER), Barcelona, Spain; 7 Institut de Recerca Biomèdica de Barcelona, Parc Científic de Barcelona, Barcelona, Spain; 8 Bioinformatics Unit, Structural Biology and Biocomputing Programme, CNIO (Spanish National Cancer Research Centre), Madrid, Spain; 9 Department of Urology, MD Anderson Cancer Center, Houston, TX USA; 10 Department of Urology, Heinrich-Heine-University, Düsseldorf, Germany; 11 University of Colorado Comprehensive Cancer Center, Aurora, CO 80045 USA; 12 Translational Genomics Laboratory, Division of Cancer Epidemiology and Genetics, National Cancer Institute, Bethesda, USA

## Erratum

Please see modification to the first erratum [1] below, in which the Grant support section should have been modified as well:

Following the publication of our recent article in BMC Genomics [2] a number of aspects were called to our attention. We have carefully reviewed the experiments reported in this manuscript, additional data from our laboratories, as well as the content in Grant support section, and would like to make the following points:

1. SW-850, included in our paper as a bladder cancer cell line, has been reported by several authors to be a pancreatic cancer cell line [2–5]. This is unlikely to be the case given that most pancreatic cancers are *KRAS*-mutant and both our analysis and a previous publication [5] indicate that the cells used are *KRAS*-wild type. However, given the controversy we recommend that these cells are not be used as bladder cancer models.
2. The Materials and Methods section of our paper indicated that the following cell lines were obtained from ATCC: 253 J, 575A, 639 V, JON, MGH-U4, SW-800, SW-1710, VM-CUB-2. However, these cultures have never been distributed by the ATCC. Therefore, they are available from us if other investigators are interested in using them.
3. It has been reported that UM-UC-2 is a T24 contaminant [6–8]. We have used fingerprinting analysis to confirm this fact and the genetic identity of the cells/DNAs used in our experiments (Table 1).

**Table 1:** SNP fingerprint analysis of the bladder cancer cell lines suffering from an “identity crisis”

Cell line	Comments	D5S818	D13S317	D7S820	D16S539	VWA	TH01	AM	TPOX	CSF1PO
VM- CUB-1 p29		11	10	8,11	11,12	18,19	9	X	8	11
VM-CUB-2 p112		11,13	12	8	9	14	7	X,Y	8,12	11,12
VM-CUB-3 p65		11	9,12	8,9	12	16	9.3	X	8	12
UM-UC-2 p264	DNA fingerprinting data, same as T24	10,12	10,12	10,11	9	17	6	X	8,11	10,12
T24 p8	ATCC	10,12	12	10,11	9	17,19	6	X	8,11	10,12

*P* passage
4. It has been reported that VM-CUB-3 is a VM-CUB-1 contaminant [8] [9, 10]. Nevertheless, our data indicate that the two cultures we used as VM-CUB-1 and VM-CUB-3 are distinct at the genomic level. Furthermore, as shown in Table 1, fingerprinting analysis clearly indicates that VM-CUB-1, VM-CUB-2, and VM-CUB-3 are different from each other. The origin of the DNA/cells in our paper was as indicated in the Material and Methods section and, therefore, investigators interested in these cells could directly address the corresponding co-authors.
  In the last few years there has been much emphasis on the need to accurately designate, identify, and characterize cancer cell lines as they are precious tools for cell biology studies [11, 12]. It is with this aim that we wish to make these comments and clarifications related to our recently published work.
5. This work was supported, in part, by the Spanish Ministry of Economy and Competitivenes (MINECO) grants Consolider ONCOBIO CSD2007-00017, SAF2011-15934-E, the Institute of Health Carlos III through the Red Temática de Investigación Cooperativa en Cáncer (RTICC)] with reference number RD12/0036/0034, which is cofunded by European Regional Development Fund (ERDF); Asociación Española Contra el Cáncer, the project UROMOL EU-FP7-201663, and National Institutes of Health grants (NIH) grants RO1-CA089715; and CA075115 and CA104106 (to D.T.).
