# Supplementary material for: The UBC-40 Urothelial Bladder Cancer cell line index: a genomic resource for functional studies
Source: BMC Genomics. 2015 May 22;16(1):403. doi: 10.1186/s12864-015-1450-3 (PMC4470036; doi:10.1186/s12864-015-1450-3)

Supplementary Figure 1

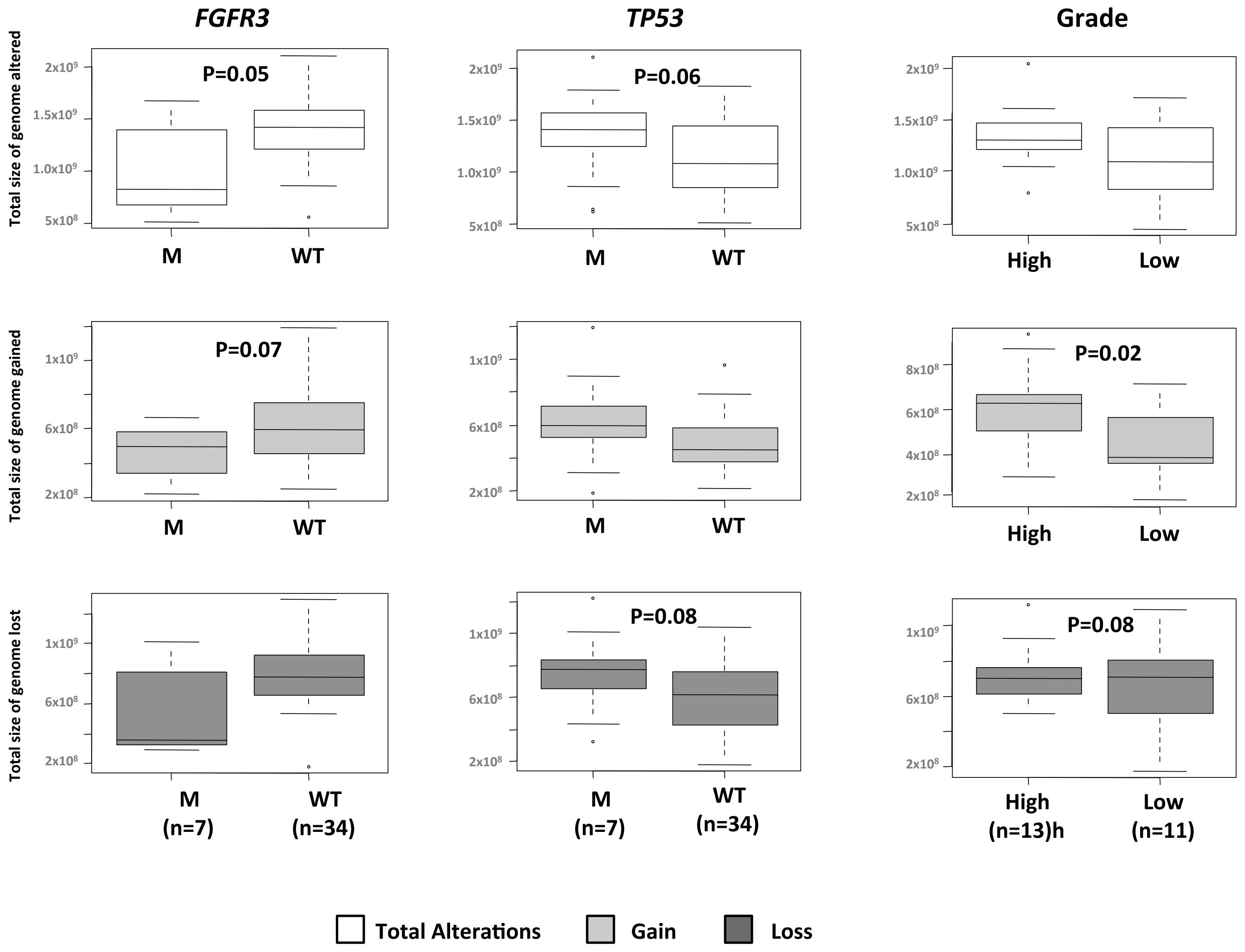

Supplementary Figure 2

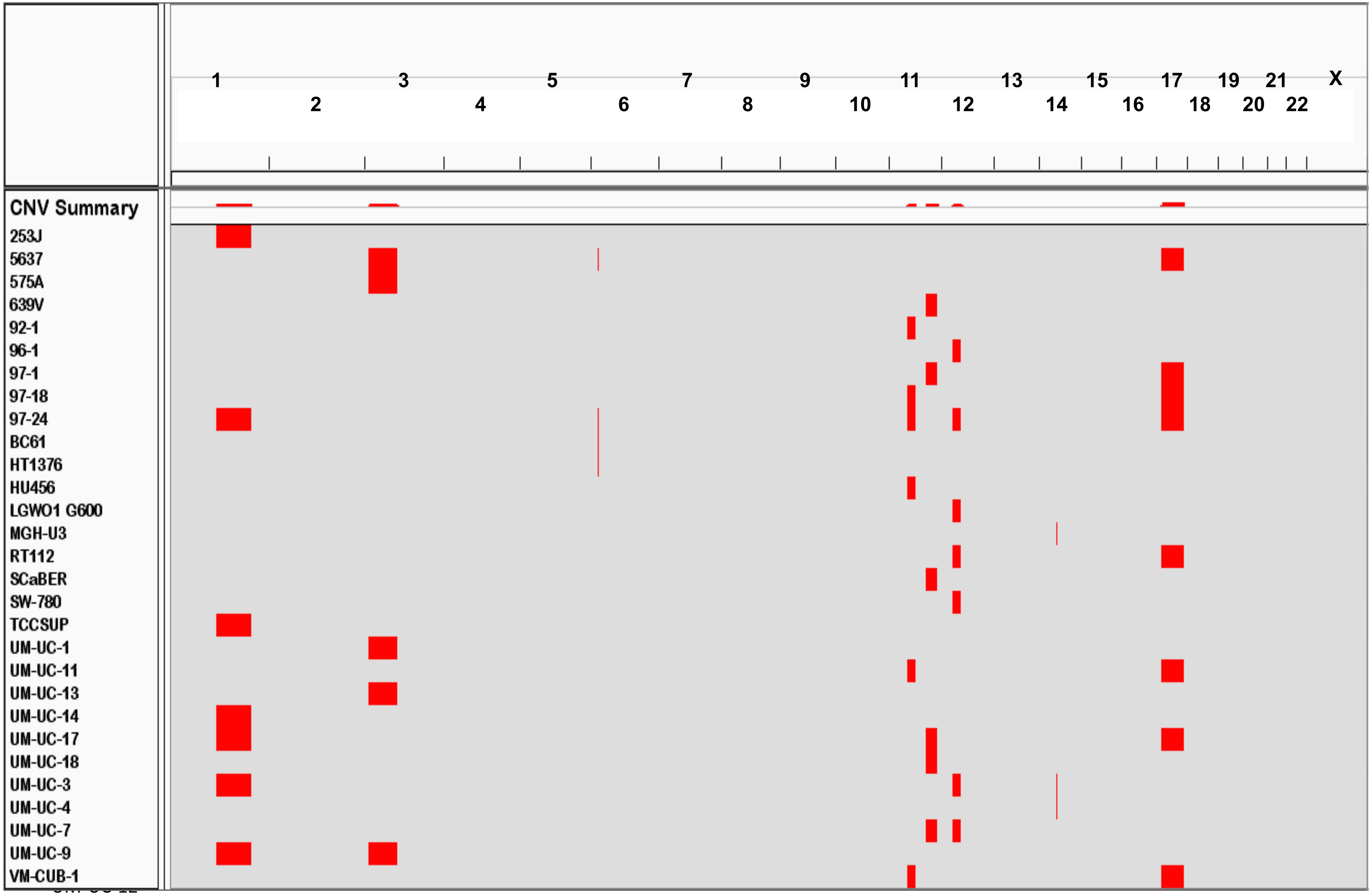

Supplementary Figure 3

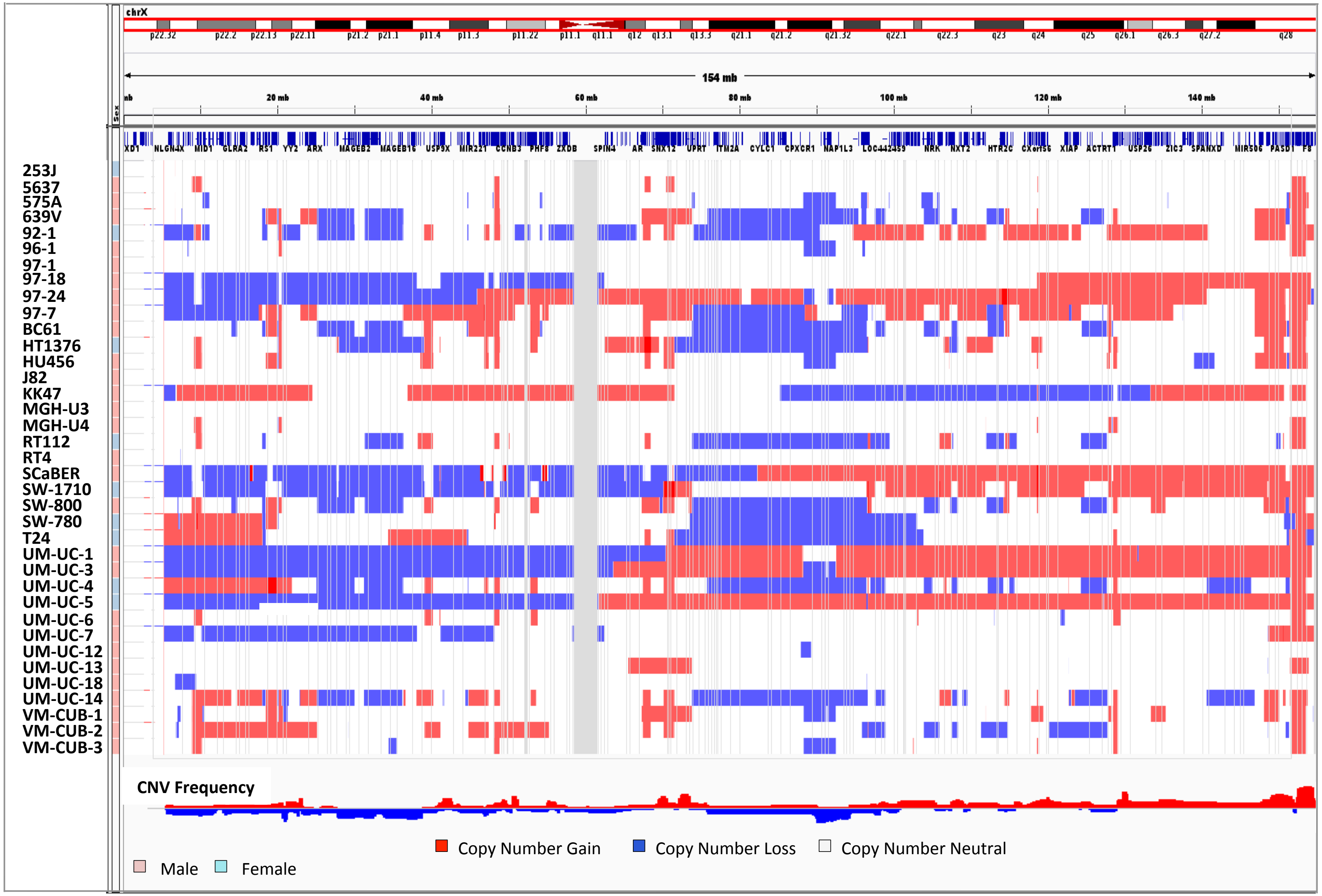

Supplementary Figure 4

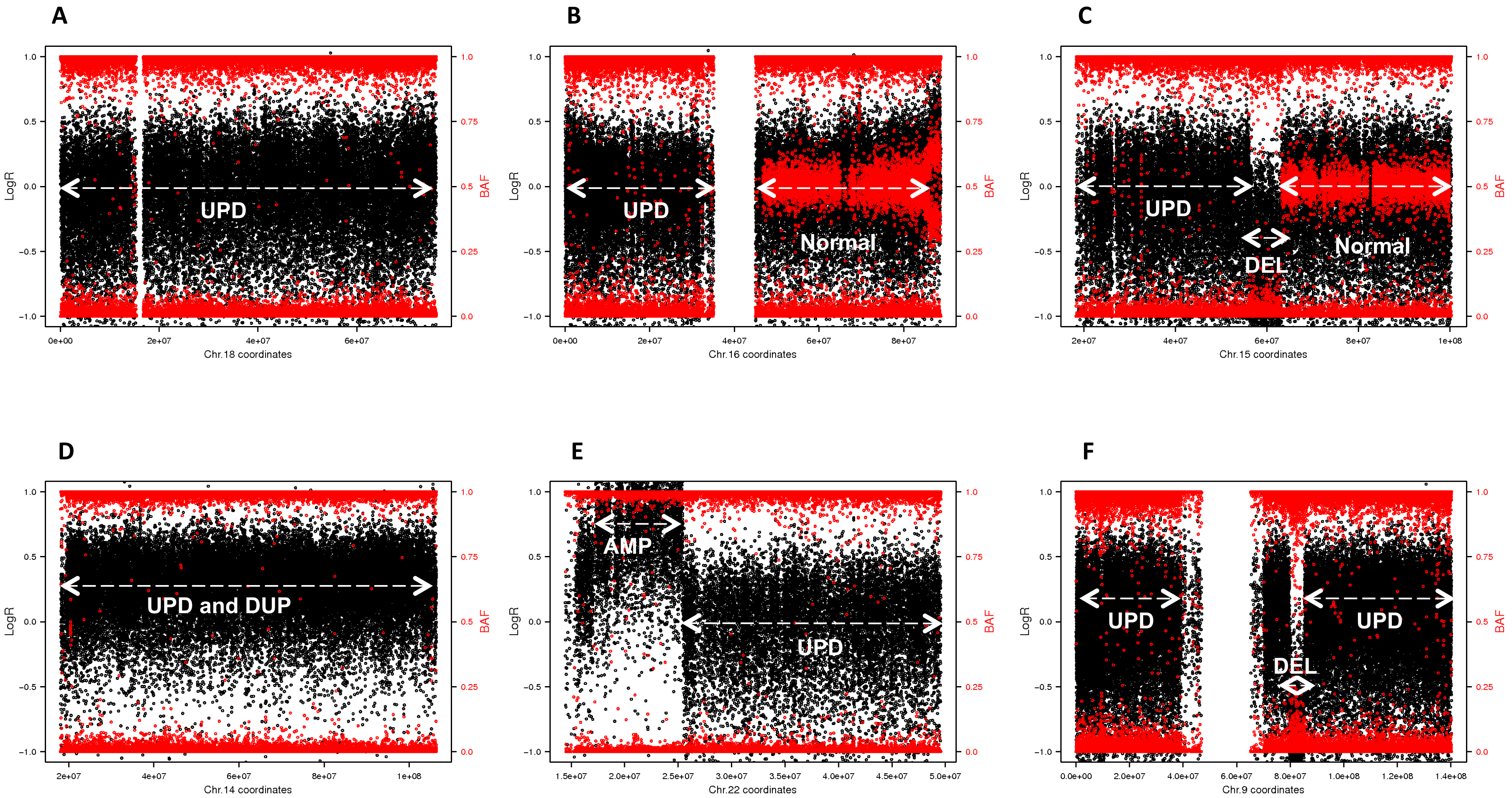

Supplementary Figure 5

A

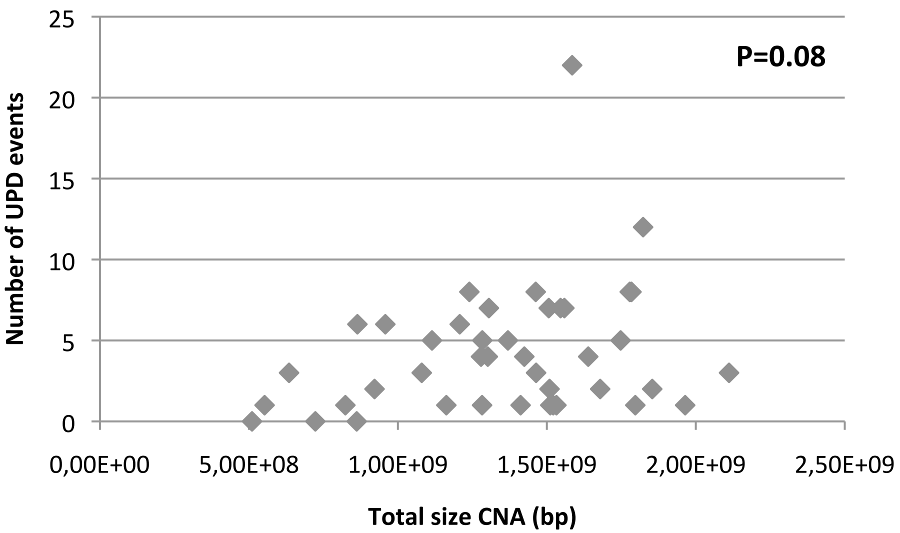

B

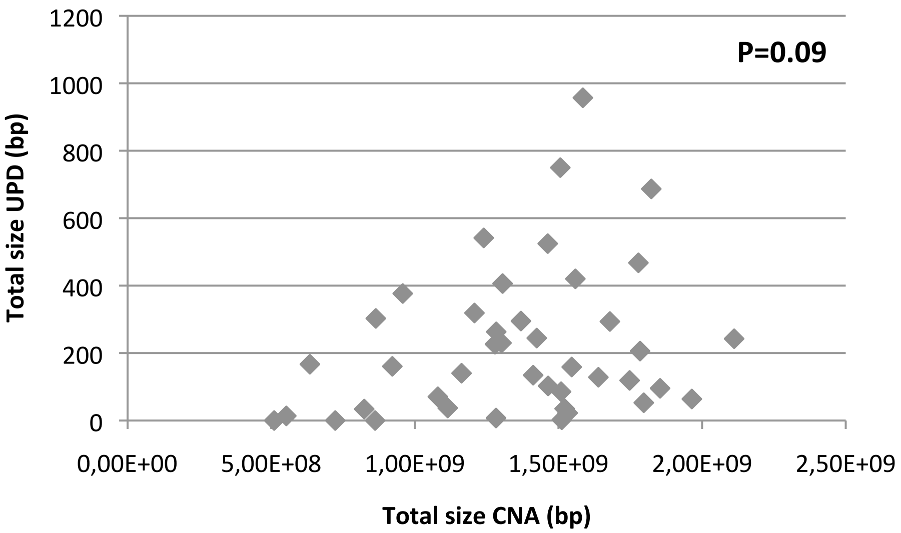

Supplementary Figure 6

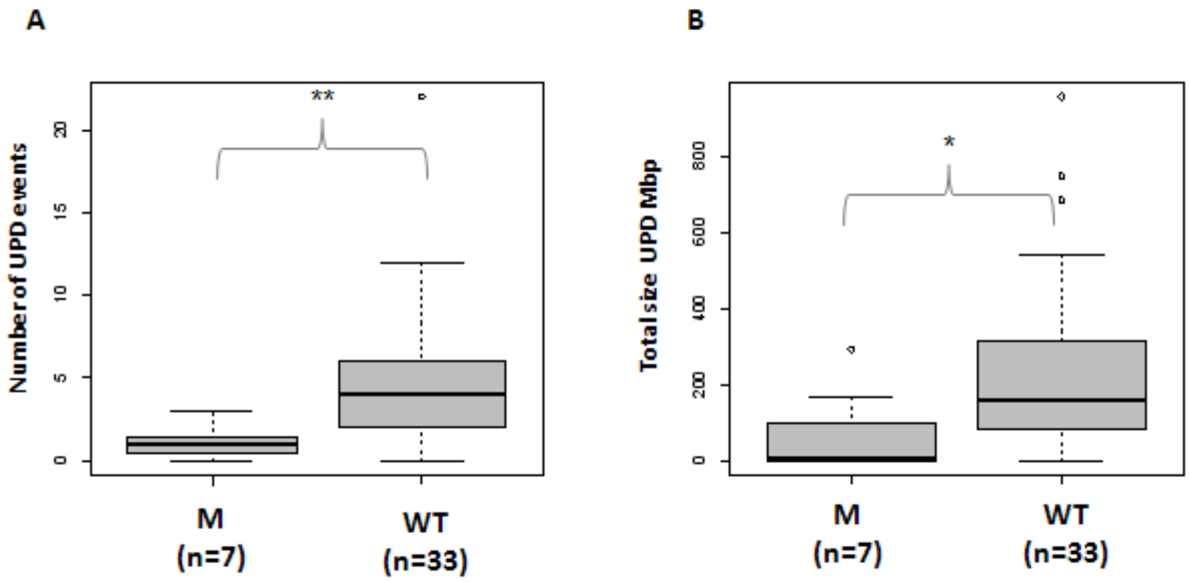

Supplementary Figure 7

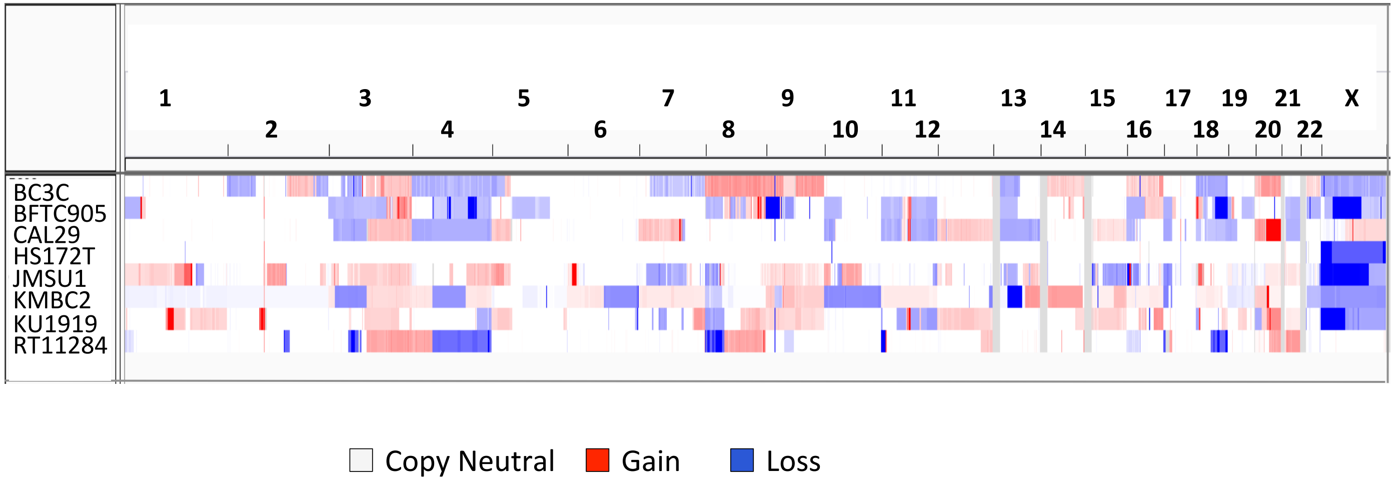

Supplement: Additional file 2: Figure S1. — Association between genomic instability (measured as fraction of the genome altered) (bp)andFGFR3 mutation status, TP53 mutation status, and Grade of original tumor from which cell lines were established. Figure S2. Genome wide assessment of gene amplifications in UBC lines. Figure S3. Log R ratios of X chromosome probe signal in UBC lines and gender origin. Figure S4. UPD analysis in UBC lines. Figure S5. Relationship between UPD events and copy number alterations. Figure S6. Uniparentaldisomies (UPD) and FGFR3 mutation status. Figure S7. Segmented mean Log ratios of probe signals for UBC lines in the Cancer Cell Line Encyclopedia (http://www.broadinstitute.org/ccle/home). [file 12864_2015_1450_MOESM2_ESM.pdf]
